# Supplementary material for: Albanol B from Mulberries Exerts Anti-Cancer Effect through Mitochondria ROS Production in Lung Cancer Cells and Suppresses In Vivo Tumor Growth
Source: Int J Mol Sci. 2020 Dec 14;21(24):9502. doi: 10.3390/ijms21249502 (PMC7764986; doi:10.3390/ijms21249502)
Supplement: Supplementary file 1 [file ijms-21-09502-s001.pdf]

Supplementary Figures for

Albanol B from mulberries exerts anti-cancer effect through mitochondria ROS production in lung cancer cells and suppresses *in vivo* tumor growth

Thanh Nam Phan[1,#], Okwha Kim[1,#], Manh Tuan Ha[2], Cheol Hwangbo[3], Byung-Sun Min[2], and Jeong-Hyung Lee[1,*]

[1]Department of Biochemistry, College of Natural Sciences, Kangwon National University, Chuncheon, Gangwon-Do 24414, Republic of Korea

[2]College of Pharmacy, Catholic University of Daegu, Gyeongbuk 38430, Republic of Korea.

[3]Division of Applied Life Science (BK21 Plus), PMBBRC, Division of Life Science, College of Natural Sciences, Gyeongsang National University, Jinju 52828, Republic of Korea

[#]These authors contributed equally to this work.

[*]Corresponding Author. Department of Biochemistry, College of Natural Sciences, Kangwon National University, Chuncheon, Gangwon-Do 24341, Republic of Korea.

Tel.:+82-33-250-8519; Fax:+82-33-259-5664; E-mail: jhlee36@kangwon.ac.kr

Running title: Anti-cancer effect of albanol B in lung cancer cells

Supplementary Figure 1.

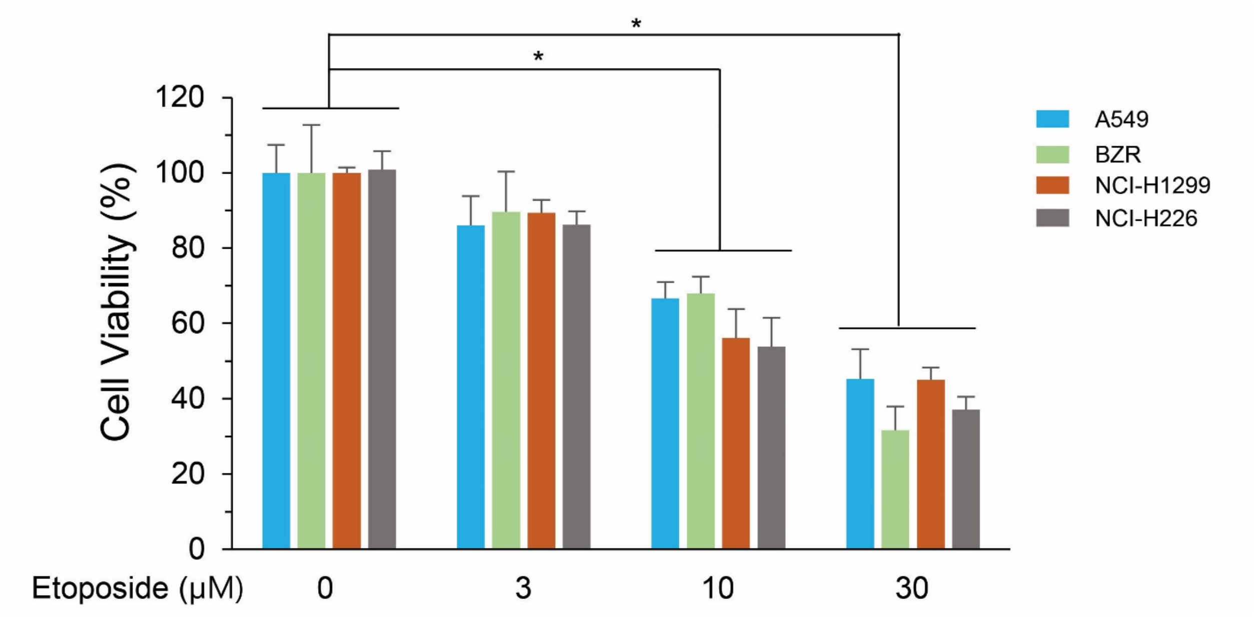

Supplementary Figure 1. Effect of etoposide on the cell viability of A549, BZR, H1975, and H226 cells. A549, BZR, H1975 and H226 cells were treated with the indicated concentrations of etoposide for 48 h. Cells viability was assessed by MTT assay. Data are presented as the mean ± SEM ($^{*}P < 0.01$ compared with vehicle-treated control; n = 3).

Supplementary Figure 2.

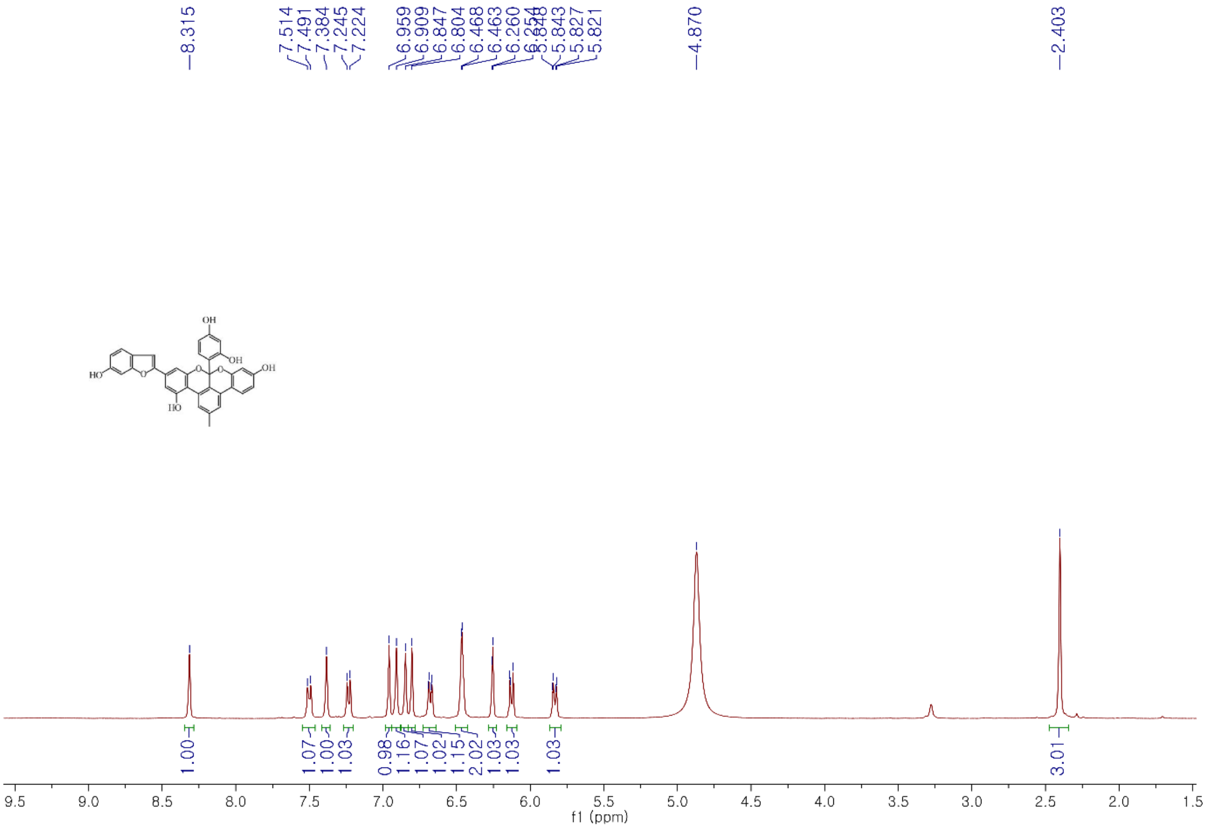

Supplementary Figure 2. ^{1}H NMR spectrum of albanol B in CD_3OD.

Supplementary Figure 3.

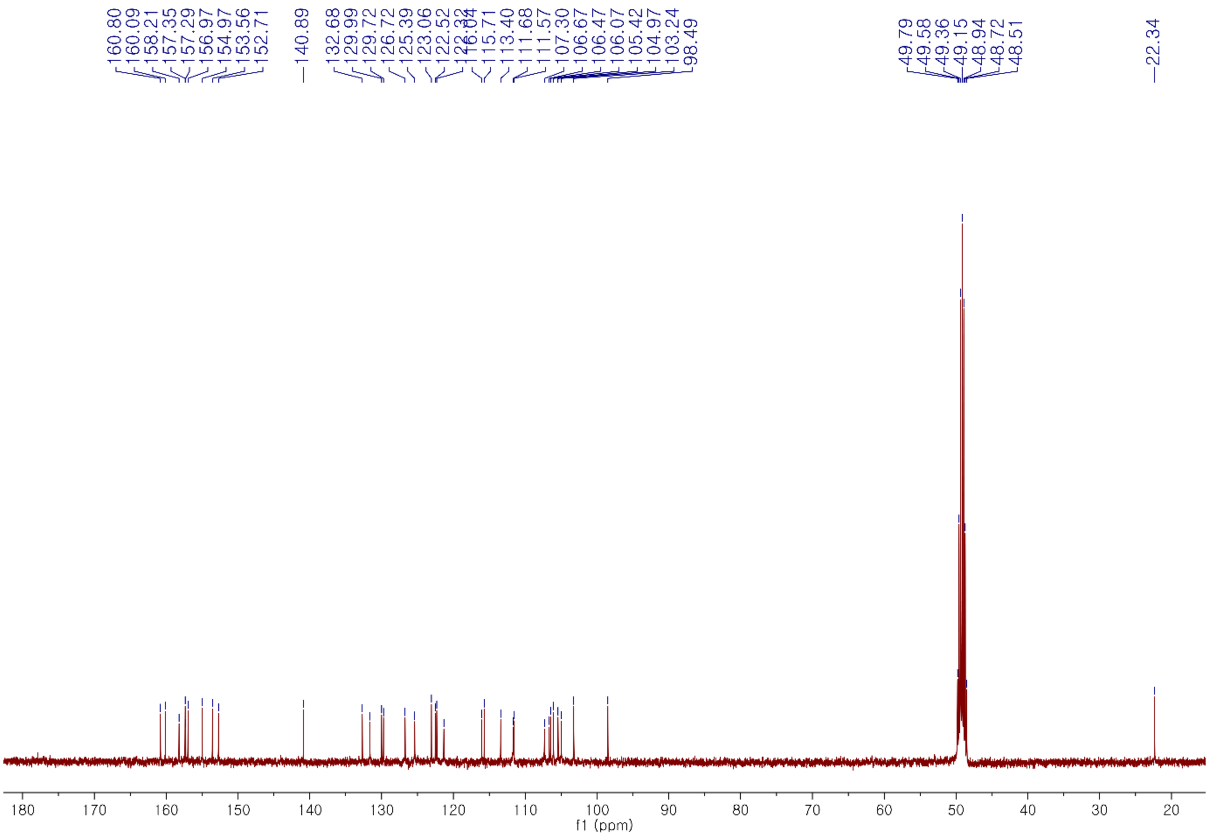

Supplementary Figure 3. ^{13}C NMR spectrum of albanol B in CD_3OD.
